# Assessing the validity of maternal report on breastfeeding counselling in Kosovo’s primary health facilities

**DOI:** 10.1186/s12884-024-06766-8

**Published:** 2024-08-27

**Authors:** Melinda McKay, Melinda K. Munos, Sunny S. Kim, Emily Bryce, Hana Bucina, Tanya Marchant

**Affiliations:** 1 Department of Disease Control, London School of Hygiene & Tropical Medicine, London, UK; 2 Institute for International Programs, Johns Hopkins University Bloomberg School of Public Health, Baltimore, MD USA; 3 Nutrition, Diets, and Health Unit, International Food Policy Research Institute, Washington, DC USA; 4 Jhpiego - an Affiliate of Johns Hopkins University, Baltimore, MD USA; 5 Action for Mothers and Children, Prishtina, Kosovo

**Keywords:** Validation, Breastfeeding, Counselling, Measurement, Indicators, Primary care, Quality of care

## Abstract

**Background:**

Improving the quality of breastfeeding counselling delivered by primary care providers can improve breastfeeding outcomes and ultimately reduce mortality and morbidity of children and mothers. Accurate data on coverage and quality of primary care breastfeeding counselling is essential for monitoring progress; however, global and national indicators are limited. To help address this gap, this study validated indicators of receipt and quality of breastfeeding counselling during routine consultations for infant care at seven primary health facilities across Kosovo.

**Methods:**

Mothers’ reports of breastfeeding counselling received during routine consultations for their infants (0–12 months of age) were collected by exit interview in 2019 and 2021 (*n* = 609). Responses were compared against direct observation of their consultation using a structured checklist (reference standard) by a trained third-party observer at the primary care facility. We assessed 13 indicators; ten were related to the receipt and content of breastfeeding counselling, and three were specific to the provider’s interpersonal skills. We calculated sensitivity, specificity, and area under the receiver operating curve (AUC) to determine individual-level reporting accuracy.

**Results:**

Ten indicators had an agreement rate above 70% and seven indicators had high overall individual-level validity (AUC ≥ 0.7). High prevalence indicators recorded high sensitivity and low specificity, and the inverse for low prevalence indicators. More subjective indicators were less reliable, e.g., mothers over-reported the prevalence of all three indicators related to providers’ interpersonal skills.

**Conclusions:**

This study offers evidence on breastfeeding counselling quality by validating maternal reports of whether a provider discussed breastfeeding, the clinical content of that counselling, and how it was delivered. It is also situated in a primary care setting within a fragile state of which there is limited evidence. We observed that mothers reported accurately when asked directly to recall breastfeeding counselling services received. However, there is a need to further validate subjective questions about interpersonal skills and other measures for the ‘experience of care’ quality dimension.

**Supplementary Information:**

The online version contains supplementary material available at 10.1186/s12884-024-06766-8.

## Background

Breastfeeding is a high impact and cost-effective health behavior; yet, in most countries it remains inadequately practiced and supported. Optimal breastfeeding practices would save the lives of 823,000 children and 20,000 mothers globally each year and provide economic gains of $300 + billion annually [1, 2]. This opportunity is especially significant for fragile and conflict affected states, where maternal, neonatal and nutritional conditions are the cause of the greatest number of Disability Adjusted Life Years (DALYs), nearly twice the amount caused by HIV/AIDS, TB and malaria combined [3]. Access to and quality of health services are key determinants of breastfeeding practices [1], and primary care providers in particular can play an important role given their frequent access to mothers and caregivers across the continuum of care (i.e., during routine antenatal, postnatal, infant care and immunization consultations) [4]. The WHO recommends at least six breastfeeding counselling contacts across these periods, and most of this care occurs in a primary health care or community setting [5]. Improving the breastfeeding counselling practices of health providers can improve breastfeeding outcomes [1, 6–10]. As breastfeeding practices rapidly deteriorate with child age [11, 12], offering better counselling and support at the primary care level after birth presents a clear opportunity to improve sustained breastfeeding practices.

Accurate data on the coverage and quality of breastfeeding counselling delivered is important to gauge progress on national and international maternal and child health goals. However, global and national monitoring of breastfeeding counselling is generally very limited. Large-scale household surveys such as the Demographic and Health Survey (DHS) [13] and Multiple Indicator Cluster Survey (MICS) [14], collect self-reported retrospective data and are often the only source of reliable data for maternal and child health service coverage and outcomes [15], even in fragile and conflict-affected states. The coverage question added to the DHS-8 about breastfeeding counselling received two days after birth does not address content or quality of counselling, nor the recommended number of breastfeeding counselling events found to be critical in the child’s first years of life [5]. The WHO’s current Global Nutrition Monitoring Framework did not include an indicator to track if mothers with young children received counselling about breastfeeding in the last 12 months, because the data to calculate it was not available in DHS until DHS-8 (data presently available in only a few countries), MICS, or national systems [16]. UNICEF’s Nutridash monitoring platform indicates that of the 57 countries reporting data, most have incorporated infant and young child feeding counselling into at least 75% of their primary care facilities, but there is no information on population coverage or quality of service [12]. Further work is needed to understand how to accurately assess breastfeeding counselling coverage and quality at all health system levels. While there is no universal definition of “quality” in this context, WHO offers guidance [5] on the content, timing, frequency and mode of counselling, and the necessary clinical and interpersonal practices to achieve quality breastfeeding counselling.

While there are a range of efforts underway worldwide to strengthen monitoring and measurement of maternal and child health, significant gaps persist in service quality and coverage indicators [17], particularly for breastfeeding counselling. Understanding the accuracy of maternal reporting on breastfeeding counselling received is important to inform how to measure and interpret that data. Furthermore, few studies situated in low- and middle-income countries have validated counselling practices in the last decade, and those that did were mostly focused on family planning [18–23]. This study aimed to validate maternal reports of health providers’ breastfeeding counselling behaviors during routine consultations with their infants aged 0–12 months in primary care facilities in Kosovo, a European middle income country and a fragile state [24].

## Methods

### Study design

A criterion validity study of maternal report of breastfeeding counselling behaviors was implemented in primary health facilities in Kosovo. Breastfeeding-friendly practices documented by trained observers during routine one-to-one consultations at the primary care health facility were used as the reference standard against which to validate mother’s reports of breastfeeding counselling received during those consultations, in accordance with recent validation research on reproductive, maternal, newborn and child health and nutrition (RMNCH + N) indicators [25]. Maternal reports were collected by exit interview immediately after the consultation. We assessed the validity for indicators at the individual level using the area under the receiver operating curve (AUC).

### Study setting and populations

This validation study was nested within a larger parent study that designed and evaluated a behavior-centered approach to improving breastfeeding-friendly practices of primary health care providers in Kosovo. The parent study’s primary outcome of interest was the average prevalence rate of breastfeeding-friendly practices observed at an individual provider level during routine consultations after the intervention compared to the prevalence before the intervention. Its secondary outcome of interest was why the intervention did, or did not, change provider behavior, assessed using a range of process indicators. The parent study generated evidence on what works and why to change primary healthcare provider practices and offered a science-based framework to identify and affect underlying drivers of behavior (see Additional File 1 for more information).

Kosovo was chosen for the study setting given its high neonatal mortality setting (four times that of the European average [26, 27]) and low and declining breastfeeding rates (29% of infants under six months are exclusively breastfed versus the global average of 44% [26, 28]). Furthermore, there is a high frequency of care provided to infants in its public primary care system, but limited research on the prevalence and quality of breastfeeding counselling has made it difficult to assess where and how to improve support.

**Fig. 1:**
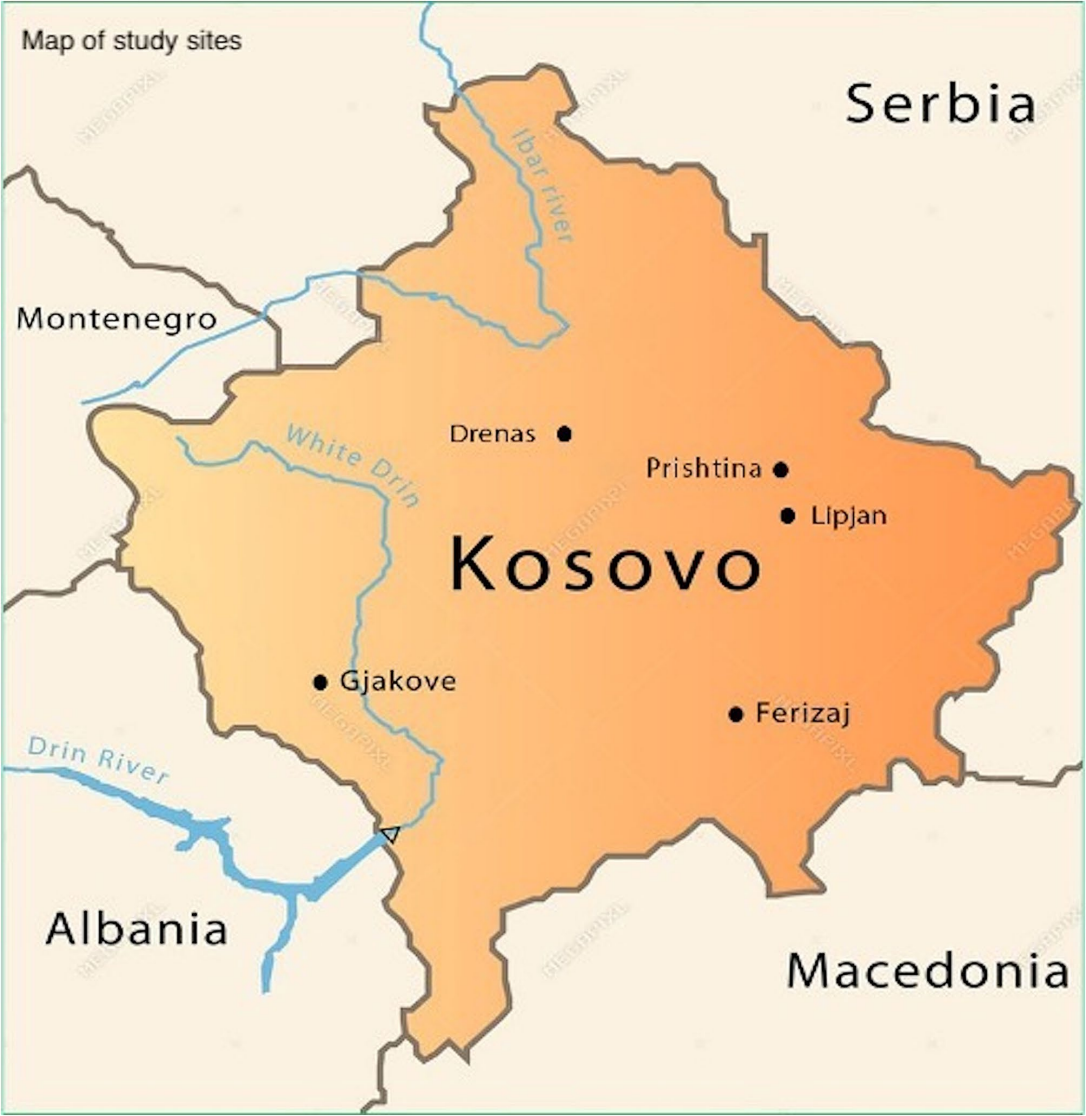
Map of study sites

At the primary care level, 29 of Kosovo’s 38 municipalities have one Main Family Medicine Center, which provides diagnostic and curative care, including antenatal and postnatal services and immunizations. Population catchment areas of 10,000 persons generally have smaller Family Medicine Centers, and in some municipalities, these are the focal point for infant immunizations. The validation research was implemented in seven Family Medicine Centers situated in five municipalities throughout the country (see Fig. 1), all in an urban setting and offering a similar range of services.

### Ethical approval and consent

Ethical approval was granted by the Hospital and University Clinical Services of Kosovo Professional Ethics Committee (#133/6240), the Kosovo Doctors Chamber, London School of Hygiene & Tropical Medicine Observational and Interventions Research Ethics Committee (#16357) and Johns Hopkins School of Public Health Institutional Review Board (#9653). Written informed consent was obtained from all participants (mothers and providers) prior to observations during the first round of data collection and changed to verbal informed consent during the second round of data collection to comply with COVID-19 safety protocols.

### Data collection

The study focused on routine consultations related to the care of infants aged 0–12 months, which included postnatal checks, acute care visits, routine care visits and immunization services. All mothers of infants who attended a consultation of interest with a provider who had consented to participate in this study were eligible for participation. Mothers were excluded if they were under 18 years of age or were unable to provide consent.

Cross-sectional data was collected at two time periods, each lasting approximately five days per site during the parent study’s baseline data collection in 2019 and endline data collection in 2021. Observers used a structured checklist (Additional files 2 and 3) to assess breastfeeding counselling delivered during routine consultation. Exit interviews with mothers were conducted immediately after the consultation in a nearby private space within the facility to limit courtesy bias, and recall erosion. Exit interviews included the identical checklist (Additional files 4 and 5) of provider-client interactions as the observation checklist. No exit interview was conducted unless the reference consultation was observed. To limit selection bias, we followed each health provider for a certain time period each day and observed all consultations with that provider within that observation period. Data was collected using paper forms before being entered into tablets for encrypted uploading to a secure server.

Extensive effort was made to culturally adapt the interview and observation tools, including indicator wording, to ensure accurate interpretation by the study sample and data collectors. Alongside a well-established local NGO focused on maternal and child health, the study contracted female Kosovar data collectors who were experienced primary care clinicians with research and breastfeeding counselling expertise and fluency in Albanian and English. They led the adaptation of the tools from English to Albanian. To ensure the original meaning was maintained for each question, the adapted tools were pretested at a facility not part of this study, as recommended in WHO guidelines [29]. This pretesting led to helpful modifications to the final tools, which were vetted by the study’s advisory board that included representatives from the Ministry of Health, WHO, UNICEF, Baby Friendly Hospital Initiative (BFHI) Kosovo committee and primary care facility leadership.

The study used Kosovar data collectors external to the study sites to minimize potential for bias. Clinicians with research and deep breastfeeding counselling expertise conducted the observations. Medical students with prior research experience conducted the exit interviews. All data collectors were female to ensure mothers and providers felt comfortable, and they were all fluent in Albanian (in which all consultations and interviews were conducted) and English. Data collectors received training in the research objectives and methods and procedures for ethical research, and use of the exit interview questionnaire and observation checklist to ensure a full and consistent understanding of how to record responses. This included classroom-based instruction and role-play. Additionally, prior to the first round of data collection there was facility-based testing at a site not included in the sample where two observers assessed the same consultation to check interobserver agreement. All data collectors underwent training before both rounds of data collection, and some data collectors were the same for both periods.

### Indicator selection

Indicators selected for validation were identified through a review of published and grey literature, and based on relevant training materials (primarily from WHO and Unicef), survey instruments and current questions in DHS and MICS household surveys [5, 30–52], to identify important components of breastfeeding counselling by primary care providers during routine primary care consultations with mothers and their children 0–12 months of age. These were adapted and prioritized in consultation with local specialists, an approach deemed “crucial” in a recent paper on definitions of indicator validity in maternal and newborn child health [53]. These are not indicators used for the global tracking of breastfeeding counselling since they were selected to measure the effect of the parent study intervention, but their validation still provides a useful contribution to the global conversations on how to collect data on breastfeeding counselling. Subsequent piloting of the data collection instruments allowed for further refining of the indicator list. A total of 13 indicators were selected for validity testing (see Table 1): ten indicators were on provision of any breastfeeding counselling and content of breastfeeding counselling (clinical behaviors), i.e., the “what”, and three indicators were specific to the interpersonal skills of health providers i.e., the “how”. For each of the ten clinical behavior indicators, prevalence is identified by the number of “yes” responses as a percentage of all responses. For each of the three interpersonal behavior indicators, prevalence is indicated by a rating of “a lot” or “a great deal” on a five-point Likert scale as a percentage of all responses, while a “moderate amount” or lower was treated as a “no” response given the courtesy bias other studies have reported when validating interpersonal dimensions of family planning counselling [18, 19, 22, 23]. We aimed to limit the subjective nature of the interpersonal skills indicators by clearly defining the scope of each indicator during the training of data collectors (Additional file 6), testing the survey instrument at a facility outside of the sample prior to the first round of data collection to assess both interobserver agreement and mothers’ interpretation of questions, and by using female Kosovar data collectors to ensure collegial and clear interpretation of the indicator.

**Table 1:** Breastfeeding counselling indicators with corresponding exit interview questions

INDICATOR	OBSERVATION/EXIT INTERVIEW QUESTION
**1. CLINICAL BEHAVIORS**	**During the consultation did the staff:**
% of primary health care providers that discussed breastfeeding or infant feeding with mothers.	Talk about any infant feeding or breastfeeding or how baby is being fed?
% of primary health care providers that explained the benefits of breastfeeding to mothers.	Explain the benefits of breastfeeding (exclusive breastfeeding under six months and/or continued breastfeeding until baby is 2 + years)?
% of primary health care providers that explained a woman’s physiological ability to breastfeed to mothers.	Explain that most women are able to breastfeed (physiological ability)?
% of primary health care providers that asked mothers if they had any breastfeeding questions or concerns.	Ask if there were any questions or concerns related to breastfeeding?
% of primary health care providers that explained follow-up visits required to mothers.	Give an explanation of follow up visits required?
% of primary health care providers that inquired about mothers’ support structure.	Ask if people around (mother/you) support her to breastfeed?
% of primary health care providers that gave take-home material to mothers.	Give any information to take home about breastfeeding?
% of primary health care providers that explained breastfeeding support resources available to mothers.	Explain where to get information/support for breastfeeding?
% of primary health care providers that did not promote breastmilk substitutes to mothers.	Promote or provide samples of breastmilk substitutes (formula)?
% of primary health care providers that observed mother breastfeeding.	Observe breastfeeding?
**1. INTERPERSONAL BEHAVIORS**	***During the consultation did the staff***:
% of primary health providers who really listened to, and understood the concerns of, mothers.	Really listen to (mother/you) and understand her concerns?
% of primary health care providers who made mothers feel comfortable to express their opinions, feelings and concerns.	Make (mother/you) feel comfortable to express her opinions, feelings and concerns?
% of primary health care providers who explained things well and gave practical help in a way mothers could understand.	Explain things well and give practical help in a way (mother/you) could understand?

### Sample size

There was little-to-no prior data on coverage of breastfeeding counselling during consultations for infants aged 0–12 months at primary care facilities in Kosovo, but consultation with local stakeholders and review of a report that included nutrition counselling practices of primary care providers in Kosovo [30] suggested that an average prevalence rate of 25% for all indicators would be reasonable. Using moderate to high sensitivity (65% or above) and specificity (75% or above), a ± 7% precision and α = 0.05 assuming normal approximation to the binomial distribution, a target sample size of 510 was calculated using Buderer’s formula [54]. All mothers attending infant consultations during the data collection period were invited to participate in the study until at least the target size of sample for each site had been reached.

### Data analysis

Data from the two time-periods were combined. To test homogeneity between the two rounds of data collection, a chi-squared test was performed on sample characteristics across the two time periods. Sensitivity analysis was conducted to compare results between the 2019 and 2021 data collection to assess possible effects of COVID-19 and the parent study intervention that aimed to improve breastfeeding counselling practices by providers. Exit interviews with mothers were held one-to-one i.e., without their companion present, in almost all cases across both rounds of data collection.

Data was analyzed using Stata17 [55]. All “don’t know” responses were excluded from the matched pairs analysis. Accuracy at the individual level was assessed by comparing the responses of mothers in the exit interviews with the reference standard (observed) response to each indicator in two-by-two tables. The receiver-operating curve was plotted for each indicator and the area under the receiver-operating curve (AUC) was calculated as a measure of average accuracy for each indicator [25]. The AUC ranges between 0 and 1, with 0.5 equivalent to a random guess and 1 a perfect measure. The benchmark of validity for this study has been set at an AUC of ≥ 0.7 [25]. A table comprising the full list of indicators selected for validity testing as well as the prevalence of each indicator as reported by mothers and observers was formulated. Within this table, the AUC was presented alongside sensitivity (SE) and specificity (SP) to demonstrate the potential reporting bias of each indicator. The rate of “don’t know” or non-reported response for each indicator was described.

## Results

### Sample description

We observed 72 health providers across a total of 647 consultations (277 and 370 from the first and second rounds, respectively), and of those consultations we conducted a paired exit interview with 98% (*n* = 637) of mothers. We excluded 28 paired observations and exit interviews from the analysis because the infant was aged 13 months or older. This resulted in a total of 609 mothers (277 and 332 from the first and second round respectively) with paired observation and exit interviews. Table 2 describes the sample characteristics for infants, mothers and providers included in the study sample. Two-thirds of consultations were with a nurse and over 90% of consultations were with a female provider. Almost all mothers identified as ethnically Albanian. Most had either secondary or higher education and 85% were aged 18–34 years. Over 60% of women had parity of 2 or more, and of those women, most (84%) breastfed their previous child(ren) for some period. Only 40% of mothers reported receiving any breastfeeding counselling during pregnancy, 74% reported receipt of breastfeeding counselling within two days of baby’s birth and 69% received additional breastfeeding counselling during the baby’s first month of life i.e., 3–30 days after birth. The primary purpose for just over half of all observed consultations was immunization, since Family Medicine Centers are the only place permitted to administer infant immunizations in Kosovo and coverage rates are high [26]. Accordingly, over half of infants were three months or younger. Less than half (45%) of all infants were being exclusively breastfed according to the mother’s report.

**Table 2:** Sample characteristics

CHARACTERISTICS: INFANT	*n* = 609	%
**Consultation type**		
Immunization	320	53%
Routine check for baby	126	21%
Acute visit for baby	158	26%
Acute visit for mother	5	1%
**Infant age**		
< 1 month	141	23%
1–3 months	183	30%
4–6 months	101	17%
7–12 months	184	30%
**Current breastfeeding status**		
Exclusive (< 6 months)	274	45%
Any (0–12 months)	178	29%
No, but previously did (0–12 months)	87	14%
Never (0–12 months)	70	11%
**CHARACTERISTICS: MOTHER**	*n* = 609	**%**
**Mother’s ethnicity**		
Albanian	581	95%
Did not answer	28	5%
**Mother’s education level**		
Primary or pre-primary	78	13%
Secondary	281	46%
Higher	239	39%
Did not answer	11	2%
**Current age of mother**		
18–24	127	21%
25–29	216	35%
30–34	175	29%
35+	87	14%
Did not answer	4	1%
**Prior breastfeeding counselling**		
During pregnancy	244	40%
During the first two days after baby’s birth	448	74%
Anytime within the first month of baby’s birth (3–30 days after birth)	421	69%
**Mother’s prior parity**		
0	236	39%
1 or more	372	61%
*Mother did breastfeed prior child/ren*	*312*	*84%*
*Mother did not breastfeed prior child/ren*	*59*	*16%*
*DId not answer*	*1*	*0%*
Did not answer	1	0%
**CHARACTERISTICS: PROVIDER**	*n* = 609	**%**
**Provider position**		
Consultations with a Nurse	393	65%
Consultations with a Midwife	1	0%
Consultations with a Doctor	215	35%
**Provider gender**		
Consultations with a Female provider	562	92%
Consultations with a Male provider	47	8%

The chi-squared test on sample characteristics across the two data collection time periods showed variation for three characteristics; 76% of mothers recalled having had prior breastfeeding counselling within the first month of baby’s birth in the second round compared to 61% in the first round (chi-2 *p*-value < 0.001); there were fewer acute care visits in the second round at 21% versus 32% of all visits in the first round (chi-2 *p*-value 0.007); and as a direct consequence of COVID-19 prevention measures, 36% of mothers were accompanied by a companion during the consultation in the second round compared to 80% in the first round (chi-2 *p*-value < 0.001).

### Validity of indicators

For each of the 13 indicators, prevalence, percentage of “don’t know” responses, percent agreement with the reference standard, sensitivity, specificity, and AUC are detailed in Table 3. Six indicators recorded a “don’t know” response of greater than 5%, but none exceeded 7%. Three indicators had an agreement rate of 90% or higher. All but one indicator (*Provider did not promote breastmilk substitutes*) had at least five counts per cell, making the chi-square test valid for all but that one indicator. Four indicators had high sensitivity (≥ 85%) and five indicators had high specificity (≥ 85%). Six of the ten clinical indicators and one of the three interpersonal indicators had high overall validity according to the study’s criteria AUC ≥ 0.7.

**Table 3:** Validation results: comparing routine consultation observations with mother’s reports at exit interviews

	Observations (Reference Standard) *N* = 609	Exit Interviews with Mothers *N* = 609		Matched Pairs						
INDICATOR	Prevalence (95% CI)	Prevalence (95% CI)	Don’t Know	Matched Pairs (*N*)	Agreement (%)	5 Counts *p*/cell	Sensitivity (95% CI)	Specificity (95% CI)	AUC* (95% CI)	AUC criteria met*
**1. CLINICAL BEHAVIORS**										
Provider discussed breastfeeding or infant feeding.	90 (88–93)	87 (84–89)	0%	609	87%	Yes	90.7 (88.0–93.0)	52.5 (39.1–65.7)	0.72 (0.65–0.78)	Yes
Provider explained the benefits of breastfeeding.	59 (55–63)	58 (54–62)	3%	569	72%	Yes	76.0 (71.1–80.5)	67.0 (60.4–73.0)	0.71 (0.68–0.75)	Yes
Provider explained a woman’s physiological ability to breastfeed.	39 (35–43)	33 (29–37)	6%	552	67%	Yes	51.6 (44.8–58.3)	77.5 (72.6–81.9)	0.65 (0.61–0.69)	No
Providers asked mother if she had any breastfeeding questions or concerns.	52 (48–57)	35 (31–37)	5%	495	71%	Yes	59.4 (53.2–65.4)	84.7 (79.4–89.1)	0.72 (0.68–0.76)	Yes
Provider explained follow-up visits required.	95 (93–97)	69 (66–73)	2%	586	72%	Yes	72.4 (68.4–76.0)	69.0 (49.2–84.7)	0.71 (0.62–0.79)	Yes
Provider inquired about mothers’ support structure.	15 (12–18)	6 (4–8)	7%	542	83%	Yes	12.8 (6.6–21.7)	95.8 (93.6–97.5)	0.54 (0.51–0.58)	No
Provider gave take-home material about breastfeeding.	11 (9–14)	9 (7–12)	6%	550	91%	Yes	51.5 (38.9–64.0)	96.1 (93.9–97.6)	0.74 (0.68–0.80)	Yes
Provider explained breastfeeding support resources available.	17 (14–20)	15 (12–18)	7%	543	83%	Yes	47.4 (37.2–57.8)	91.3 (88.2–93.7)	0.69 (0.64–0.75)	No
Provider did not promote breastmilk substitutes (formula).	96 (94–97)	98 (97–99)	7%	541	94%	No	98.1 (96.5–99.1)	3.8 (0.1–19.6)	0.51 (0.47–0.55)	No
Provider observed mother breastfeeding.	14 (12–17)	14 (11–17)	6%	553	90%	Yes	63.1 (51.9–73.4)	94.5 (92.0-96.3)	0.79 (0.73–0.84)	Yes
**2. INTERPERSONAL BEHAVIORS**										
Provider really listened to, and understood the concerns of, mother.	38 (35–42)	74 (71–78)	2%	586	53%	Yes	86.3 (81.1–90.5)	32.5 (27.7–37.6)	0.59 (0.56–0.63)	No
Provider made mother feel comfortable to express her opinions, feelings and concerns.	39 (35–43)	73 (70–77)	2%	584	54%	Yes	85.5 (80.3–89.8)	33.4 (28.5–38.6)	0.59 (0.56–0.63)	No
Provider explained things well and gave practical help in a way mother could understand.	30 (26–34)	42 (38–46)	2%	585	72%	Yes	72.3 (65.1–78.8)	71.3 (66.7–75.7)	0.72 (0.68–0.76)	Yes

*AUC = area under the receiver operating characteristic curve. AUC criteria for high reporting accuracy: AUC ≥ 0.7.

Sensitivity analysis by year of data collection (Additional file 7) revealed patterns that were consistent with the larger, combined dataset, although exit interviews recorded higher prevalence rates for all indicators in 2021 compared to 2019, while observers recorded higher prevalence rates for 11 of the 13 indicators; and the AUC was higher for all indicators in 2021 compared to 2019.

### Clinical behaviors

Two indicators (*Provider discussed breastfeeding* and *Provider did not promote breastmilk substitutes*) had very high prevalence according to both observer and mother reports, at 87% and 98% respectively. Four indicators (*Provider inquired about mother’s support structure*,* Provider gave take-home material about breastfeeding*,* Provider explained breastfeeding support resources available*,* and Provider observed mother breastfeeding*) had low prevalence according to both observer and mother reports, ranging from 6 to 17%. For all clinical behaviors, agreement between the two responses was 67% or higher. In all but one indicator (*Provider did not promote breastmilk substitutes*) mothers’ reported prevalence was lower than the reference standard and for three indicators the difference was substantial, with no overlap in the confidence interval (CI) between observer and mother. Mothers reported a 26-percentage point (pp) lower prevalence for *Provider explained follow up visits required* (95% CI for Observer | Mother: 93–97 | 66–73), a 17-pp lower prevalence for *Provider asked mother if she had any breastfeeding questions or concerns* (95% CI Observer | Mother: 48–57 | 31–37) and a 9-pp lower prevalence for *Provider inquired about mothers’ support structure* (95% CI for Observer | Mother: 12–18 | 4–8).

High individual-level reporting accuracy was registered for six of the ten clinician behavioral indicators: *Provider discussed breastfeeding or infant feeding* (SE 90.7%, SP 52.5%), *Provider explained the benefits of breastfeeding* (SE 76.0%, SP 67.0%), *Provider asked mother if she had any breastfeeding questions or concerns* (SE 59.4%, SP 84.7%), *Provider explained follow-up visits required* (SE 72.4%, SP 69.0%), *Provider gave take-home material about breastfeeding* (SE 51.5%, SP 96.1%) and *Provider observed mother breastfeeding* (SE 63.1%, SP 94.5%). While not meeting the AUC or minimum cell-count criteria due to insufficient variation in responses, the indicator *Provider did not promote breastmilk substitutes* had a 94% agreement rate with the reference standard (SE 98.1%, SP 3.8%). High specificity and moderate to low sensitivity were recorded for the three other indicators that did not meet the AUC criteria: *Provider explained a woman’s physiological ability to breastfeed* (SE 51.6%, SP 77.5%), *Provider inquired about mothers’ support structure* (SE 12.8%, SP 95.8%) and *Provider explained breastfeeding support resources available* (SE 47.4%, SP 91.3%).

### Interpersonal behaviors

Mothers over-reported the prevalence of all three interpersonal indicators between 12- to 36-percentage points compared with the reference standard. Two interpersonal indicators that relate to providers listening to mothers and making them feel comfortable, recorded the study’s lowest agreement rates at 54% each and low specificity (32.5% and 33.4% respectively). One interpersonal indicator (*Provider explained things well and gave practical help in a way mother could understand*) met the study’s AUC criteria for high individual level accuracy. Sensitivity rates were moderately high across all three indicators, ranging from 72.3 to 86.3%, while specificity was only moderately high for the indicator assessing if provider explained things well (71.3%).

## Discussion

This study of breastfeeding counselling behaviors validated women’s responses about the provider-client interaction immediately after the consultation in Kosovo using a criterion validation approach. Overall, we found high agreement rates for the indicators, with a few exceptions, and high validity for more than half of the indicators. This suggests that, in general, mothers could accurately report the counselling services received. Consistent with other studies [18, 19, 22, 23], indicators that are more subjective were found to be less reliable with two of the three interpersonal indicators having agreement rates just over 50% and an AUC of 0.59. These results add to the small but growing research validating RMNCH + N counselling coverage and quality in low- and middle-income settings.

Our findings of overall high validity contrast with those of many other criterion validation studies of labor and delivery care that have found generally poor validity of women’s responses about interventions received [21, 56–61], with all but one of these studies using exit interviews for mothers’ report. There was one exception in the Kenya/Eswatini antenatal and postnatal care study for the breastfeeding counselling indicator (provider discussed breastfeeding/feeding for baby), which yielded high agreement rates, along with moderately high sensitivity and specificity [21]. The study in China hypothesized recall can be influenced by the “clinical and cultural context” [62]. Our study’s setting (primary care facility) and consultation type (routine/acute infant care, so not in the intrapartum or immediate postnatal period) might account for the better performance; the hypothesis being that recall is lower during stressful and/or intense periods. Results in other studies with these contexts are mixed. Three studies, set in (i) China, (ii) Bangladesh/Cambodia/Kenya, and (iii) Haiti/Malawi/Senegal, offered insights into provider practices during routine antenatal care [18, 62, 63]. These found generally poor recall and validity across counselling indicators. The study in China surmised that when population coverage of an intervention is high there is better client recall during surveys [62]. Similarly, a recent study on delivery and newborn care in Nepal [59] and another study on family planning service quality in Cambodia and Kenya [22] concluded the same; over-reporting of more common interventions or methods, and under-reporting of less common interventions or methods. This is consistent with our study, where population level coverage of breastfeeding counselling within two days of birth is 81.9% [26].

Criterion validation studies have been extensively used for health indicators, particularly for measures of whether a particular intervention was delivered [53], because these indicators often seek to measure the “true” care received by an individual – for example, the diagnostics, medical treatment, and/or advice provided – in order to prioritize interventions, design and evaluate health programs, and identify under-served populations [25, 64, 65]. Understanding the extent to which measured coverage indicators accurately reflect “true” population coverage is therefore relevant and of interest to many RMNCH + N practitioners and researchers. While criterion validation approaches have been widely used to assess the accuracy of reporting of MNCH&N indicators, they do have limitations, particularly with regard to the establishment of an objective “truth” or reference standard. In this study, we used a criterion validation approach to understand how maternal reports of breastfeeding counselling and interpersonal behaviors compared to those of trained observers. While we made efforts to standardize the observation of clinical and interpersonal behaviors, we acknowledge that our reference standard, like all reference standards, was imperfect and not necessarily a reflection of the mother’s experience of care. Criterion validity is only one way of examining the validity of these indicators - there are several other approaches that could be used, including examining construct validity (of which criterion validity contributes), as well as more qualitative approaches such as cognitive interviewing. An important topic for future research is to examine these indicators, particularly for interpersonal behaviors, using other types of validation approaches.

Few criterion validation studies have validated RMNCH + N interpersonal indicators, in part because these are subjective due to differing interpretations by each reporting party and thus are inherently difficult to reliably quantify. But given the core tenant of quality breastfeeding counselling is to give equal emphasis to what providers say and how they say it [52], validating interpersonal dimensions of counselling is an important yet neglected area of study [22]. For that reason, there is interest in understanding how to measure interpersonal behaviors in a way that produces data that are useful to decision-makers. Our findings are consistent with four other studies that validated interpersonal family planning counselling behaviors which recorded a range of agreement rates and generally low specificity compared with sensitivity. These studies assessed family planning service quality and included: validation of exit interviews with primary care consultation observations in Cambodia and Kenya [22]; validation of Service Provision Assessment (SPA) survey results with DHS data in Haiti, Malawi, Senegal and Tanzania [19]; a similar study comparing SPA survey results with DHS data in Haiti, Malawi, Senegal [18]; and, a study that compared provider interviews, client interviews, and observations of client-provider interactions in Kenya [23]. Our results add to this body of evidence finding that that low performance of counselling indicators could be explained by courtesy bias, whereby clients tend to over-report receiving care and under-report negative practices. This is consistent with Kosovo’s paternalistic culture and the normative doctor-patient power dynamic. Gaining a better understanding of discrepancies in the validity of maternal reporting of interpersonal indicators could help to inform decisions about whether and how to collect these data, and can provide insight into how to improve the effectiveness of the interactions between providers and clients (mothers).

This study made efforts to reduce observer subjectivity and improve the reference standard through a detailed scoring checklist adapted from WHO and Unicef training materials [5, 37, 45, 47, 49–52] (Additional file 6), training and supervision during data collection. More information on the data collection approach of other studies involving direct observation of clinical care would be useful to increase sophistication and consistency of measurement. While emerging literature has commented on the value of using criterion validity methods in the context of behavior and counseling indicators [66], criterion validation methods continue to be well described and recommended for this type of study [25].

In contrast to over-reporting service receipt of interpersonal behaviors, mothers under-reported receiving services for most of the measures of clinical behaviors in this study. This was the case even for the more objective measures of receiving take-home material, whether the provider explained follow up visits required and if the mother had any questions about breastfeeding. We hypothesize this might be because the providers weren’t using ‘shqip të thjeshtë’ (plain Albanian) but the observers, as senior doctors themselves, could understand the provider’s messages. It could also be a function of the relatively low quality of interpersonal skills as ‘objectively’ [22] measured by the observations recorded by physicians (not to imply mother’s perception of care received is less ‘true’) but there is insufficient evidence to confirm this hypothesis. Since some indicators assessed in this study are experiential, it could be the mothers’ experience of the care was more influential than what actually happened. This is consistent with other studies [18, 22, 63].

The second round of data collection occurred in the context of the COVID-19 pandemic which might have affected provider behaviors and subsequently mother’s recall during the exit interview. Additionally, an intervention to improve breastfeeding counselling practices of providers was conducted by the parent study between the first and second round of data collection. Given the results of the sensitivity analysis, the authors hypothesize that the quality improvement intervention and COVID-19 acted as opposing positive-negative forces on the prevalence of breastfeeding-friendly counselling behaviors and maternal recall, but this cannot be measured with certainty. The data collectors reported that in 2021 providers in the study sites were experiencing a type of COVID-induced apathy due to additional work demands and task-shifting. While this might have been expected to affect prevalence rates, it’s unlikely to have impacted sensitivity and specificity since it would have affected observer and mother responses alike. It’s possible that the intervention designed to increase breastfeeding-friendly provider practices helped to increase prevalence despite COVID-19’s described impact on providers and mothers, but this conclusion is limited by the parent study’s before-after design.

The strength of this study is that it validates not just whether a provider discussed breastfeeding, but the clinical content of that counselling and how it was delivered i.e., interpersonal behaviors. It is also situated in a primary care setting within a middle-income, fragile state and to our knowledge, it is the first to do so in these contexts. Only four of the 17 papers validating RMNCH + N practices in low- and middle-income countries published in the last decade were situated in a fragile or conflict-affected setting [18–20, 67], none were conducted in Europe and many studies were in a hospital setting. Our study adds to the body of evidence validating maternal, newborn, and child care content and quality [53]. The study returned overall high validity, with six indicators registering an AUC of at least 0.7 and a lower limit of between 0.62 and 0.73 of the 95% CI. We used observations as the reference standard, and while this is a strength, observations can also be subject to error from incorrect observer interpretation or documentation. In addition, the Hawthorne effect could impact provider behavior but it unlikely to have affected the accuracy of maternal recall. Courtesy bias is more likely to have impacted maternal reporting of interpersonal behaviors. Social desirability bias is a possible explanation for the reported high rates of exclusive breastfeeding for this sample (45%) versus the national average of 29% [26], with mothers potentially over-reporting the desirable behavior of breastfeeding. The short time between the visit and exit interview likely renders stronger validity than for questions with longer recall periods such as those included in the DHS or MICS, where mothers are asked to recall events some time after they occurred. While recall erosion is not an issue in this study, recall bias is a possible limitation since the mother might not have had a chance to process all the messaging she received. A similar issue was hypothesized in a study validating family planning quality of care in Ecuador, Uganda and Zimbabwe [20].

Capturing mothers’ experience of care, the subjective aspect of counseling quality, is important to improve the interactions between providers and clients (mothers). If mothers are not able to understand, recall or relate to what the health provider communicates then they are unable to translate that advice into improved breastfeeding practices, which impacts both child and mother health. Conversely, if the health provider is unaware that their messaging is not translating to mothers in an effective way, they cannot improve their own counselling practices. Information about the accuracy of maternal reporting on the counselling they received and how that counselling is delivered is needed to inform decisions about how to measure breastfeeding counselling and how to interpret counselling data. This study aimed to address multiple evidence gaps and respond to the call for additional effort for improved coverage of RMNCH + N service quality indicators [17].

## Conclusion

Breastfeeding has a substantial positive impact on child and mother mortality and morbidity, and accurately measuring the coverage and quality of counselling delivered by providers is essential to inform improvement efforts. In this study to validate key aspects of breastfeeding counselling we found that mothers with young children who visited primary care facilities were able to provide valid responses during exit interview about the breastfeeding counselling they received, although more subjective indicators had less reliability. While these results are encouraging for tracking progress in Kosovo, the subjectivity inherent in measuring the quality of counselling are sufficiently important to justify further research to strengthen their measurement.

### Electronic supplementary material

Below is the link to the electronic supplementary material.

Supplementary Material 1

Supplementary Material 2

Supplementary Material 3

Supplementary Material 4

Supplementary Material 5

Supplementary Material 6

Supplementary Material 7

## Data Availability

The data that support these findings and the following supplementary material include: Additional file 1: Background on the parent study. Additional File 2: Client Exit Interview Questionnaire 2021 (English). Additional file 3: Client Exit Interview Questionnaire 2021 (Albanian). Additional file 4: Observation Checklist 2021 (English). Additional file 5: Observation Checklist 2021 (Albanian). Additional file 6: Observation Scoring Guide. Additional file 7: Comparison of validation results for the two sample periods.

## Abbreviations

AUC: Area under the receiver-operating curve
CI: Confidence interval
DALYs: Disability Adjusted Life Years
DHS: Demographic and Health Survey
MICS: Multiple Indicator Cluster Survey
RMNCH + N: Reproductive, Maternal, Newborn and Child Health and Nutrition
SE: Sensitivity
SP: Specificity
SPA: Service Provision Assessment
WHO: World Health Organization
