# Supplementary material for: Assessing the validity of maternal report on breastfeeding counselling in Kosovo’s primary health facilities
Source: BMC Pregnancy Childbirth. 2024 Aug 27;24:558. doi: 10.1186/s12884-024-06766-8 (PMC11348650; doi:10.1186/s12884-024-06766-8)
Supplement: Supplementary file 1 — Supplementary Material 1 [file 12884_2024_6766_MOESM1_ESM.pdf]

Additional File 1: Background on the parent study

STUDY DESIGN AND THEORY OF CHANGE

The study aimed to measure breastfeeding counseling coverage and test the effectiveness of a creative intervention to sustainably increase breastfeeding-friendly practices in Kosovo's primary care facilities. The objectives were two-fold:

1. Undertake formative research to build context-specific local knowledge about determinants of provider behavior to inform the design of a pilot intervention.
2. Conduct a cross-sectional evaluation to measure breastfeeding counseling coverage before and after the pilot intervention to assess any change in the practices of relevant health providers and determine feasibility for scale-up of the pilot intervention.

The study sought to answer the following questions:

1. What are the current breastfeeding-friendly practices of primary care providers?
2. What are the levels of technical knowledge and interpersonal skills of primary care providers?
3. What are the motivations for primary care providers to provide breastfeeding support?
4. What are the current constraints to implementing breastfeeding-friendly practices at primary care facilities and provider ideas to improve practices?
5. How do actual breastfeeding-friendly practices by primary care providers compare with mother's reports of breastfeeding counseling received during private consultations at the facility?
6. Was there an improvement in breastfeeding-friendly counseling practices by providers after the pilot intervention compared with before the pilot intervention?
7. Why, or why not, did the pilot intervention change primary care provider behavior?

The abridged hypothesized pathway to change of the intervention is described in Figure 1.

Figure 1: Theory of Change

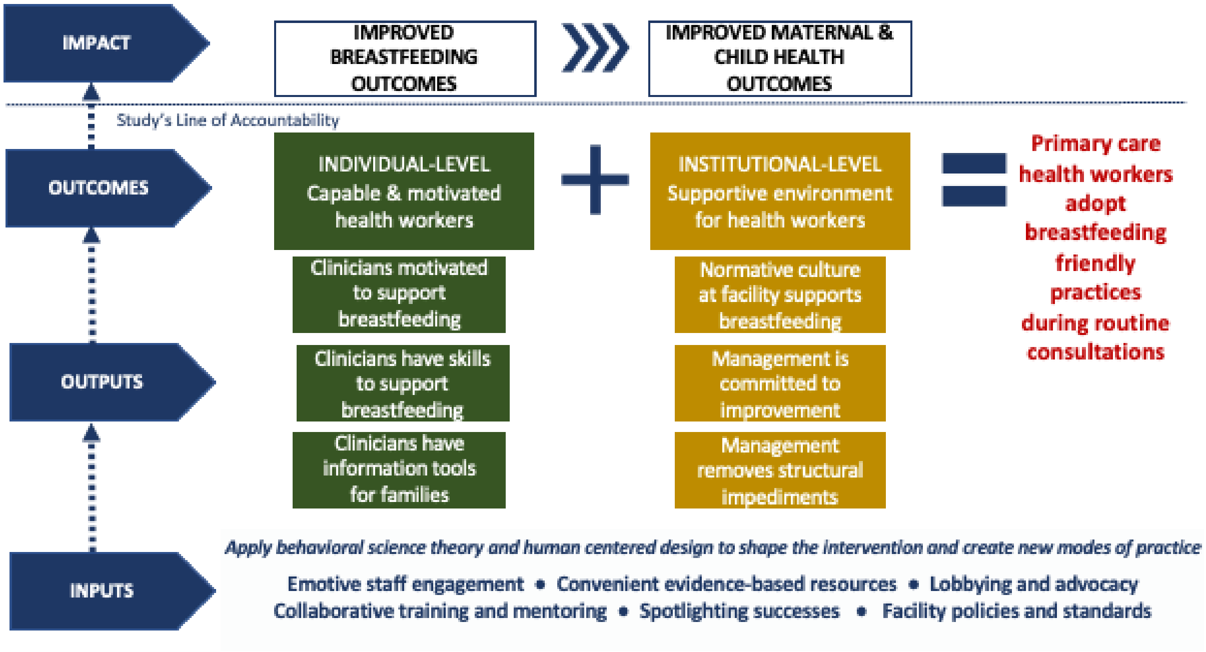

INTERVENTION DESIGN

A mixed methods approach was adopted. It commenced with a 'framing workshop' with key stakeholders to discuss findings from the Literature review (which identified barriers/facilitators to breastfeeding friendly practice), the current situation in Kosovo, research design, any potential impacting factors and to hypothesize how to influence behavior. Data collected during 2019 in the primary care facilities was used in the formative research and baseline evaluation. This was informed by the literature and framing workshop insights and was situated in the behavior setting. It included site observations, clinical observations, client exit interviews, provider one-on-one interviews and provider focus groups. After findings were collated, 'insight generation workshops' with key stakeholders revised the preliminary theory of change and generated a creative brief for the design of a pilot intervention. These design thinking workshops applied Behavior Centered Design-related tools including Touchpoint Mapping and Barrier/Facilitator Validation. Synthesis of these insights generated a list of potential interventions. These were then ranked with an Intervention Component Ranking Tool to ensure selection of the most appropriate intervention activities.

INTERVENTION DESCRIPTION

A multifaceted intervention targeted to affect individual behaviors and the institutional environment was implemented. The primary focus was on affecting motives by linking the desired behavior with *status* and *affiliation* (highly respected and modern clinicians support breastfeeding). It also targeted executive and reactive brain mechanisms. The intervention was paused midstream, adapted, then resumed 12 months later due to COVID-19. While delaying the research, the result was greater clinician/leader engagement in intervention design e.g. Photovoice, and more scalable activities e.g. clinical APP and online training. Contact was maintained during pause with leaders but not clinicians due to the Government of Kosovo mandates. The activities implemented by the Kosovar NGO Action for Mothers and Children between 2019 and 2021 included:

- **Motive engagement:** 1) Provider away-day 2) Direct messaging to providers on closed Viber groups 3) Social and mainstream media exposure 4) Photovoice 5) Motivational videos 6) Online training
- **Props and tools:** 7) Posters 8) Brochures 9) Clinician APP
- **Institutional change:** 10) Leadership advocacy 11) Breastfeeding pledge that featured an adaptation of WHO/UNICEF's Baby Friendly Hospital Initiative "Ten Steps to Successful Breastfeeding"[1].

INTERVENTION EVALUATION

Data was then collected in 2021 for use in the endline evaluation. A 'results workshop' with key stakeholders debated the findings and assessed viability for scale-up.

[1] Protecting, promoting and supporting breastfeeding in facilities providing maternity and newborn services: implementing the revised Baby-friendly Hospital Initiative 2018. Geneva: World Health Organization and the United Nations Children's Fund (UNICEF), 2018. Licence: CC BY-NC-SA 3.0 IGO.
