# Supplementary material for: Assessing the validity of maternal report on breastfeeding counselling in Kosovo’s primary health facilities
Source: BMC Pregnancy Childbirth. 2024 Aug 27;24:558. doi: 10.1186/s12884-024-06766-8 (PMC11348650; doi:10.1186/s12884-024-06766-8)
Supplement: Supplementary file 2 — Supplementary Material 2 [file 12884_2024_6766_MOESM2_ESM.pdf]

Additional file 2 - Client Exit Interview Questionnaire 2021 (English)

Background: This validation study was nested within a larger parent study that designed and evaluated a behavior-centered approach to improving breastfeeding-friendly practices of primary health care providers in Kosovo. Therefore the questionnaire asks questions outside the scope of the validation study. The full questionnaire for the parent study endline data collection (2021) is presented below.

DO NOT ASK MOTHER THESE QUESTIONS:

1. Date: ______ 2. FMC number: ______ 3. Patient number: ______ 4. Staff number: ______

5. Interviewer name: __________ 6. Interview duration: ____ minutes (start: __________ end: __________)

7. Visit type: __ postnatal check mother __ postnatal check baby __ immunization visit baby
__ routine check baby __ acute care baby __ acute care mother __other

8. Baby's age: __ months 9. Others present: __yes (who: _____________) __no

---

START OF INTERVIEW

10. What is your age? ____(years)

11. What is the highest level of school you have attended?
__pre-primary __primary __lower secondary __upper secondary ___higher

12. To what ethnic group do you belong? __Albanian __Serbian __Other __Did not answer

13. Do you have any other children? __ yes __no

14. If yes, were they ever breastfed? __ yes __ no

15. How would you rate your overall experience at the FMC today? *(show response card #1)*
1 = very bad, 2 = bad, 3 = average, 4 = good, 5 = very good, 6 = don't know/didn't answer
1 2 3 4 5 6

16. During your consultation today did the healthcare provider:
1= yes, 2=no, 3=don't know, 4=N/A

a.	Talk about infant feeding or breastfeeding or how your baby is being fed?	1	2	3	4
b.	Explain the benefits of breastfeeding (exclusive BF under six months or continued BF until baby is 2+ years)	1	2	3	4
c.	Explain that most women are able to breastfeed (physiological ability)	1	2	3	4
d.	Ask if you had any questions or concerns related to breastfeeding	1	2	3	4
e.	Tell you about where to get information/support for breastfeeding	1	2	3	4
f.	Give you any information to take home about breastfeeding	1	2	3	4
g.	Ask if people around you support you to breastfeed	1	2	3	4
h.	Promote or provide samples of breastmilk substitutes	1	2	3	4

i. Give you an explanation of follow up visits required 1 2 3 4
j. Observe you breastfeeding 1 2 3 4

17. During your consultation today do you feel like the healthcare provider: *(show response card #2)*
1 = not at all, 2 = a little, 3 = a moderate amount, 4 = a lot, 5 = a great deal, 6 = didn't answer/don't know
a. Really listened to you and understood your concerns 1 2 3 4 5 6
b. Made you feel comfortable to express your opinions, feelings and concerns 1 2 3 4 5 6
c. Explained things well and gave practical help in a way you could understand 1 2 3 4 5 6

18. Before today, did a health care provider or community worker talk with you about breastfeeding:
1= yes, 2=no, 3=don't know/didn't answer/not applicable
a. During your pregnancy 1 2 3
b. During the first two days after baby's birth 1 2 3
c. Anytime within the first month of baby's birth (3-30 days after birth) 1 2 3

19. Do you currently breastfeed your baby?
__ yes exclusive __ yes mixed (BM + formula/other) __ no but previously did __ no never

20. Do you believe that you can successfully breastfeed your baby? *(show response card #2)*
1 = not at all, 2 = a little, 3 = a moderate amount, 4 = a lot, 5 = a great deal, 6 = don't know/not applicable
1 2 3 4 5 6

21. After your consultation today have your feelings changed about: *(show response card #2)*
1 = not at all, 2 = a little, 3 = a moderate amount, 4 = a lot, 5 = a great deal, 6 = don't know or N/A
a. The value of breastfeeding for your baby's health 1 2 3 4 5 6
b. The length of time you will breastfeed your baby 1 2 3 4 5 6
c. Your ability to overcome breastfeeding challenges (current or future) 1 2 3 4 5 6

END OF INTERVIEW

22. Other comments/observations
(Interviewer: 1) specifically note if the mother gives her opinion on the impact of COVID-19 on her breastfeeding experience, including if she feels like she had more or less counselling/support at the FMC before or after birth, and/or at the hospital during the birth period. 2) Try to record any interesting 'quotes' from mothers about breastfeeding support at the FMC.)

____

____

____

____

____
