# Supplementary material for: Assessing the validity of maternal report on breastfeeding counselling in Kosovo’s primary health facilities
Source: BMC Pregnancy Childbirth. 2024 Aug 27;24:558. doi: 10.1186/s12884-024-06766-8 (PMC11348650; doi:10.1186/s12884-024-06766-8)
Supplement: Supplementary file 3 — Supplementary Material 3 [file 12884_2024_6766_MOESM3_ESM.pdf]

Additional File 3 - Client Exit Interview Questionnaire 2021 (Albanian): Mjeti i intervistimi në dalje

Background: This validation study was nested within a larger parent study that designed and evaluated a behavior-centered approach to improving breastfeeding-friendly practices of primary health care providers in Kosovo. Therefore, the questionnaire asks questions outside the scope of the validation study. The full questionnaire for the parent study endline data collection (2021) is presented below.

MOS I PYESNI NËNËS KËTO PYETJE:

1. Data e Intervistës: ______ 2. Numri i QMF-së:______ 3. Numri i pacientit: ______ 4. Numri i stafit: ______

5. Emri i intervistuesit: __________ 6. Kohëzgjatja e intervistës: ____ minuta

7. Lloji i vizitës: __ kontroll pas lindjës-nëna __ kontroll pas lindjës-fëmiu __ vizitë për vakcinim të fëmiut
__ vizitë rutinore-fëmiu/foshnja __ kujdes akut-fëmiu __ kujdes akut-nëna __tjetër

8. Mosha e fëmiut: __ muaj 9. Persona të tjerë prezent: __po (kush: ____________) __jo

---

START OF INTERVIEË

10. Sa vjec jeni?____(vitet)

11. Cili është niveli më i lartë i shkollimit që keni arritur?
__para-shkollor __fillore __i mesëm i ulët __i mesëm i lartë __i lartë

12. Cilit grup etnik i perkisni? __Shqiptar __Serb __Tjetër __Nuk është përgjigjur

13. A keni ndonjë fëmijë tjetër? __ po __ jo

14. Nëse po, a i keni ushqyer ata me gji?__ po __ jo

15. Si do ta vlerësonit eksperiencën tuaj në QMF sot? *(tregoni kartën e përgjigjes #1)*
(1 = shumë keq, 2 = keq, 3 = mesatare, 4 = mirë, 5 = shumë mirë, 6 = nuk e di/nuk është përgjigjur)
1 2 3 4 5 6

16. Gjatë konsultimit tuaj sot ofruesi i sherbimit shëndetësorë
(*1= po, 2=jo, 3=nuk e di, 4=nuk është përgjigjur)*

Ju ka treguar për ushqyerjen e foshnjes apo gjidhëninen apo se si është duke u ushqyer fëmiu juaj?	1 2 3 4
Ju ka treguar benefitet e gjidhenies (per gjidhenien ekskluzive nën gjashtë muaj ose vazhdimin e saj deri sa femiu mbush 2+ vjet)	1 2 3 4
Ju ka shpjeguar se shumica e grave janë në gjendje të ushqejne me gji femiun e tyre (aftësinë fiziologjike te gruas)	1 2 3 4
Ju ka pyetur se a keni ndonjë pyetje apo shqetësim rreth gjidhënies	1 2 3 4
Ju ka treguar se ku mund te gjeni infromata/mbeshtetje rreth gjidhenies	1 2 3 4

Ju ka dhënë ndonjë infromacion (broshurë) në lidhje me ushqyerjen me gji që ju ta merrni në shtëpi	1 2 3 4
Ju ka pyetur se a ju mbeshtesin në të ushqyerit me gji personat që keni në rrethin tuaj	1 2 3 4
Ju ka nxitur ose ju ka ofruar mostra për të përdorur si zëvendësim të qumështit të gjirit (p.sh. qumësht formule)	1 2 3 4
Ju ka treguar për vizitat e ardhshme që duhet t'i bëni	1 2 3 4
Ju ka shiquar se si jepni gji	1 2 3 4

17. Gjatë konsultimit tuaj sot mendoni se ofruesi i shërbimit shëndetësor: *(tregoni kartën e përgjigjeve #2)*
(1= aspak, 2 = pak, 3 = konsiderueshëm, 4 = shumë, 5 = tepër shumë, 6 = nuk e di/nuk është përgjigjur)

Me të vërtetë ju dëgjoi dhe kuptonte shqetësimet e juaja	1 2 3 4 5 6
Ju ka bërë të ndjeheni rehat për të treguar lirshëm mendimet, ndjenjat dhe shqetësimet tuaja	1 2 3 4 5 6
Ju shpjegoi gjërat mirë dhe ju dha ndihmë praktike që ju t'a kuptoni më mirë	1 2 3 4 5 6

18. Para konsultimit tuaj sot, a ka biseduar me ju rreth gjidhënies ndonjë ofrues i shërbimit shëndetësorë apo punëtorë të komuniteti: (*1= po, 2=jo, 3= nuk e di/nuk është përgjigjur/nuk aplikohet*)

Gjatë shtatëzanisë suaj	1 2 3
Gjatë dy ditëve të para paslindjes	1 2 3
Kur do gjatë muajit të parë pas lindjes (3-30 ditë)	1 2 3

18. A e ushqeni me gji momnetalisht fëmiun tuaj?
__ po ekskluzivisht __ po përzieshëm (QGJ + formula/tjetër) __ jo por më herët e kam ushqyer __ jo kurr

20. A besoni se mund ta ushqeni me gji femiun tuaj në mënyrë të suksesshme? *(tregoni kartën e përgjigjeve #2)*
(1 = aspak, 2 = pak, 3 = konsiderueshëm, 4 = shumë, 5 = tepër shumë, 6 = nuk e di/nuk është përgjigjur)
1 2 3 4 5 6

21. Pas konsultimit të sotëm a keni ndryshuar mendimin tuaj për: *(tregoni kartën e përgjigjeve #2)*
(1 = aspak, 2 = pak, 3 = konsiderueshëm, 4 = shumë, 5 = tepër shumë, 6 = nuk e di/nuk është përgjigjur)

Rëndësinë që ka të ushqyerit me gji për shëndetin e fëmiut tuaj	1 2 3 4 5 6
Kohezgjatjen që duhet ta ushqeni fëmiun tuaj	1 2 3 4 5 6
Aftësinë tuaj për të tejkaluar sfidat e gjidhënies (tani ose në të ardhmen)	1 2 3 4 5 6

FUNDI I INTERVISTËS

22. Komente të tjera/observimet: *(intervistuesi: 1) mbani sidomos shënime nëse nëna e jep mendimin e saj për ndikimin e COVID-19 në përvojën e saj me gjidhënien, përfshini nëse ajo ka patur më pak apo më shumë këshillime/përkrahje në QMF para apo pas lindjes, dhe/ose në spital gjatë periudhës së lindjes. 2) Përpiquni të shënoni ndonjë 'citim' interesant nga nënat rreth përkrahjes për gjidhënie nga QMF-ja.)*

____

____
