# Supplementary material for: Assessing the validity of maternal report on breastfeeding counselling in Kosovo’s primary health facilities
Source: BMC Pregnancy Childbirth. 2024 Aug 27;24:558. doi: 10.1186/s12884-024-06766-8 (PMC11348650; doi:10.1186/s12884-024-06766-8)
Supplement: Supplementary file 4 — Supplementary Material 4 [file 12884_2024_6766_MOESM4_ESM.pdf]

## Additional file 4 - Observation Checklist 2021

*Background: This validation study was nested within a larger parent study that designed and evaluated a behavior-centered approach to improving breastfeeding-friendly practices of primary health care providers in Kosovo. Therefore, the checklist asks questions outside the scope of the validation study. The full checklist for the parent study endline data collection (2021) is presented below. Note Q1 j. and n. were combined to provide a composite score for the indicator "Provider explained the benefits of breastfeeding".*

-----------------------------------------------------------------------------------------------------------------------

Q.1 Date: ______ Q.2 FMC number: ______ Q.3 Patient number: ______ Q.4 Staff number: ______

Q.5 Staff position: __Dr __Nurse __Midwife Q.6 Staff sex: __F __M Q.7 Observer name: _________________

Q.8 Obs. duration: ____ minutes (start: _____ end: _____ ) Q.9 Baby's age: ____ (months) __don't know

Q.10 Is mother currently breastfeeding: __yes __no __don't know

Q.11 Visit type: __postnatal check for mother __postnatal check for baby __immunization visit for baby
__routine check for baby __acute visit for baby __acute visit for mother __other

Q.12 Other staff present: __yes (__Dr __Nurse __Midwife) __no

Q.13 Was anyone with the mother: __yes (__Husband __Child __Grandmother __Other) __no

-----------------------------------------------------------------------------------------------------------------------

O.1 Clinical Skills *(1 = yes, 2 = yes but gave incorrect/incomplete information, 3 = no, 4 = N/A)*
*(Observer: For breastfeeding mothers of babies <6m check questions a-s; For breastfeeding mothers of babies >6m check questions a-m for non-breastfeeding mothers check a-c+t-u.*
*To consistently assess consultations, use the guide provided in the Survey Manual, May 2021)*

| | | | | | |
|---|---|---|---|---|---|
| | ***All mothers:*** | | | | |
| **a.** | **Talk about infant feeding or breastfeeding or how baby is being fed?*** | **1** | **2** | **3** | **4** |
| **b.** | **Give her an explanation of follow up visits required*** | **1** | **2** | **3** | **4** |
| c. | Request the Child Health Booklet *(only for routine infant care visits)* | 1 | 2 | 3 | 4 |
| | ***All breastfeeding mothers:*** | | | | |
| d. | Ask how breastfeeding was going? | 1 | 2 | 3 | 4 |
| **e.** | **Ask if mother had any questions or concerns related to breastfeeding*** | **1** | **2** | **3** | **4** |
| f. | Answer questions or explain/demonstrate how to address concerns | 1 | 2 | 3 | 4 |
| **g.** | **Ask mother if people around her support her to breastfeed*** | **1** | **2** | **3** | **4** |
| **h.** | **Explain that most women are able to breastfeed (physiological ability)*** | **1** | **2** | **3** | **4** |
| i. | Explain how to store and use expressed breastmilk | 1 | 2 | 3 | 4 |
| **j.** | **Talk about complementary feeding / benefits of continued breastfeeding*** | **1** | **2** | **3** | **4** |
| **k.** | **Give mother any information to take home about breastfeeding*** | **1** | **2** | **3** | **4** |
| **l.** | **Tell mother about where to get information/support for breastfeeding*** | **1** | **2** | **3** | **4** |
| **m.** | **Promote or provide samples of breastmilk substitutes*** | **1** | **2** | **3** | **4** |
| | ***Breastfeeding mothers with babies under six months*** | | | | |
| **n.** | **Talk about exclusive breastfeeding and explain benefits*** | **1** | **2** | **3** | **4** |
| o. | Explain importance of a good latch and show different breastfeeding positions | 1 | 2 | 3 | 4 |
| **p.** | **Observe mother breastfeeding*** | **1** | **2** | **3** | **4** |
| q. | Explain responsive feeding (feeding cues, unrestricted frequency/length) | 1 | 2 | 3 | 4 |
| r. | Explain how to know if baby is getting enough milk | 1 | 2 | 3 | 4 |
| s. | Explain alternate ways of feeding baby e.g. cup, expression | 1 | 2 | 3 | 4 |
| | ***Non-Breastfeeding mothers*** | | | | |
| t. | Ask mother if she had ever breastfed her baby | 1 | 2 | 3 | 4 |

u. Explain the value of breastfeeding and offer to help restart 1 2 3 4

**Will be validated with patient exit interview*

O.2 Interpersonal Skills *(1 = not at all, 2 = a little, 3 = a moderate amount, 4 = a lot, 5 = a great deal)*

a. Greet the mother warmly and show interest in her and her baby 1 2 3 4 5
b. Give the mother the chance to ask questions and didn't seem hurried 1 2 3 4 5
c. Practice other supportive non-verbal communication 1 2 3 4 5
**d. Really listen to the mother and understand her concerns* 1 2 3 4 5**
e. Act in a respectful and considerate way 1 2 3 4 5
**f. Make the mother feel comfortable to express opinions/feelings/concerns* 1 2 3 4 5**
**g. Explain things well and give practical help in a way she could understand* 1 2 3 4 5**
h. Reassure the mother and give her confidence 1 2 3 4 5
i. Make suggestions that did not seem like orders 1 2 3 4 5

**Will be validated with patient exit interview*

O.3 Change in engagement when infant feeding discussed *(1 = more engaged, 2 = less engaged, 3 = no)*

a. Staff 1 2 3 b. Mother 1 2 3

O.4 General observations *(Observer, specifically note if the interaction between staff and mother appears affected by COVID-19 i.e., Does mask-wearing and physical distancing protocols impact interpersonal communication? Are staff adapting to compensate e.g. are they doing anything different to pre-COVID times? Should staff have observed breastfeeding but did not? Do mothers appear less receptive to messages or assistance?)*

____________________________________________________________
____________________________________________________________
____________________________________________________________
____________________________________________________________
____________________________________________________________
____________________________________________________________
____________________________________________________________
____________________________________________________________
____________________________________________________________
____________________________________________________________
____________________________________________________________
____________________________________________________________
