# Supplementary material for: Assessing the validity of maternal report on breastfeeding counselling in Kosovo’s primary health facilities
Source: BMC Pregnancy Childbirth. 2024 Aug 27;24:558. doi: 10.1186/s12884-024-06766-8 (PMC11348650; doi:10.1186/s12884-024-06766-8)
Supplement: Supplementary file 5 — Supplementary Material 5 [file 12884_2024_6766_MOESM5_ESM.pdf]

Additional file 5 - Observation Checklist 2021 (Albanian): Mjeti i observimit: Konsultimet pas lindjes

Background: This validation study was nested within a larger parent study that designed and evaluated a behavior-centered approach to improving breastfeeding-friendly practices of primary health care providers in Kosovo. Therefore, the checklist asks questions outside the scope of the validation study. The full checklist for the parent study endline data collection (2021) is presented below.

Q.1 Data: ______ Q.2 Numri i QMF-së: ______ Q.3 Numri i pacientit: ______ Q.4 Numri i stafit: ______

Q.5 Pozita e stafit: __Dr __Infermiere __Mami Q.6 Gjinia e stafit: __F __M Q.7 Emri i observuesit: _______________

Q.8 Kohëzgjatja e observimit: ____ minutes (filloi: _____ mbaroi: _____) Q.9 Mosha e foshnjës/fëmiut: ____ (muaj) __nuk e di

Q.10 A është nëna momentalisht duke ushqyer fëmiun me gji: __po __jo __nuk e di

Q.11 Lloji i vizitës: __ kontroll pas lindjës-nëna __ kontroll pas lindjës-fëmiu __ vizitë për vakcinim të fëmiut
__ vizitë rutinore-fëmiu __ kujdes akut-fëmiu __ kujdes akut-nëna __tjetër

Q.12 Kishte staf tjetër që ishte prezent: __po (__Dr __Infermiere __Mami) __jo

Q.13 A ishte nëna e shoqëruar nga dikush: __po (__burri __fëmiju__gjyshja __tjetër) __jo

---

Q.14 Aftësitë klinike *(1 = po, 2 = po por ishin gabim/informata gjysmake, 3 = jo, 4 = Nuk është pergjigjur)*
Për nënat që ushqejnë fëmiun me gji <6m shiqoni pyetjet a-s; Për nënat që ushqejnë fëmiun me gji >6m shiqoni pyetjet a-m për nënat që nuk ushqejnë fëmiun me gji shiqoni pyetjet a-c+t-u:

**Për të gjitha nënat:**	
a. I ka treguar për ushqyerjen e foshnjës ose gjidhënien ose ka biseduar për mënyrën e ushqyerjes së fëmiut të gruas*	1 2 3 4
b. I ka shpjeguar për vizitën e rradhës që duheni t'a bëjë*	1 2 3 4
c. Ka kërkuar fletoren/kartelen shëndetësore të fëmiut (vetëm për vizitat e foshnjeve)	1 2 3 4
**Për të gjitha nënat që ushqejnë fëmiun me gji:**	
d. I ka pyetur se si është duke shkuar gjidhënia*	1 2 3 4
e. I ka pyetur se a kanë ndonjë pyetje apo shqetësim rreth gjidhënies*	1 2 3 4
f. I është përgjigjur pyetjeve ose ju ka shpjeguar se si t'i adresojnë shqetësimet	1 2 3 4
g. E ka pyetur nënën se a e mbështesin në të ushqyerit me gji personat që ka në rrethin e saj*	1 2 3 4
h. Diksutoi për aftësinë fizilogjike të gruas për të ushyqer me gji*	1 2 3 4
i. I ka shpjeguar mënyrën e ruajtjes dhe përdorimit të qumështit të gjirit	1 2 3 4
j. Ka biseduar për ushqimin plotësues si dhe për përfitimet e ushqyerjes të vazhdueshme me gji	1 2 3 4
k. I ka dhënë nënës ndonjë infromacion (broshurë) në lidhje me ushqyerjen me gji që ajo ta merr në shtëpi*	1 2 3 4
l. I ka treguar nënës se ku mund te gjejë infromata/mbeshtetje rreth gjidhenies*	1 2 3 4
m. E ka nxitur ose I ka ofruar mostra për të përdorur si zëvendësim të qumështit të gjirit (psh. qumësht formule)*	1 2 3 4
**Vetëm për nënat që ushqejnë me gji fëmiun nën gjashtë muaj:**	
n. E ka pyetur nënën se a është duke e ushqyer femiun eksluzivisht me gji dhe i ka shpjeguar benefitet*	1 2 3 4

o. I ka shpejguar rëndësinë e puthitjes të mirë dhe i ka treguar pozicione të ndryshme që mund të merr gjatë gjidhënies	1 2 3 4
p. E ka shiquar se si jep gji*	1 2 3 4
q. I ka shpjguar të ushqyerit ushqyerjen sipas kërkeses (mëkimet, frekuenca/koha e zjgatjes e pakufizuar)	1 2 3 4
r. I ka shpjeguar se si mund t'a dijë nëse fëmiu i saj ëashtë duke thither sasi të mjaftueshme të qumështit	1 2 3 4
s. I ka shpjeguar mënyra alternative për t'a ushqyer fëmiun e saj psh me filxhan, pompë	1 2 3 4
**Për nënat që nuk ushqejnë fëmiun me gji**	1 2 3 4
t. E ka pyetur se a e ka ushqyer fëmiun e saj ndonjëherë me gji	1 2 3 4
u. I ka shpejguar për rëndësinë e të ushqyerit me gji dhe i ka ofruar t'i ndihmojë të fillojnë	1 2 3 4

**Do të vërtetohet me intervistën në dalje të pacientit*

Q.15 Aftësitë ndërpersonale *(1 = aspak, 2 = pak, 3 = konsiderueshëm, 4 = shumë, 5 = tepër shumë)*

a. Ka përshëndetur guran ngrohtësisht dhe ka treguar interesim për të dhe fëmiun e saj	1 2 3 4 5
b. I dha mundësinë gruas t'i bënte pyetje dhe nuk dukej se nxitonte	1 2 3 4 5
c. Praktikoi forma të tjera të mbështetjes, jo verbale	1 2 3 4 5
d. Me të vërtetë dëgjoi gruan dhe kuptonte shqetësimet e saj	1 2 3 4 5
e. Është sjellur me respekt dhe konsideratë	1 2 3 4 5
f. E ka bërë gruan të ndihet rehat për të treguar lirshëm mendimet, ndjenjat dhe shqetësimet e saj*	1 2 3 4 5
g. Ju shpjegoi gjërat mirë dhe ju dha ndihmë praktike që ajo t'a kuptonte më mire*	1 2 3 4 5
h. E siguroi gruan për veprimet dhe i dha vetëbesim	1 2 3 4 5
i. I dha sugjerime që nuk tingëllonin si urdhëra	1 2 3 4 5

**Do të vërtetohet me intervistën në dalje të pacientit*

Q.16 Ndryshimi në përfshirjen kur të ushqyerit e foshnjës/fëmiut u diskutuan
(1 = përfshirje të madhe, 2 = përfshirje të vogël, 3 = nuk kishte ndryshim, 4 = nuk kishte përgjigje pasi që nuk u diskutua rreth mënyrës së ushqimit të fëmiut)

a. Stafi 1 2 3 4 b. Gruaja 1 2 3 4

Q.17 Observimet gjenerale
(Vëzhguesi: mbani sidomos shënime nëse ndërveprimi ndërmjet pjesëtarit të stafit dhe nënës duket se është ndikuar nga COVID-19 p.sh., a ndikon mbajtja e maskës dhe protokollet e distancimit fizik tek komunikimi ndërpersonal? A po përshtatet stafi për të kompensuar, pra a po bëjnë dika ndryshe në krahasim me kohërat para COVID-it? A është dashur stafi ta vëzhgojë gjidhënien, por nuk e kanë bërë? A ju duket se nënat janë më pak të hapura ndaj mesazheve apo ndihmës?)

____

____

____

____

____

____

____
