# Supplementary material for: Assessing the validity of maternal report on breastfeeding counselling in Kosovo’s primary health facilities
Source: BMC Pregnancy Childbirth. 2024 Aug 27;24:558. doi: 10.1186/s12884-024-06766-8 (PMC11348650; doi:10.1186/s12884-024-06766-8)
Supplement: Supplementary file 6 — Supplementary Material 6 [file 12884_2024_6766_MOESM6_ESM.pdf]

Additional file 6 – Observation Scoring Guide

Background: This validation study was nested within a larger parent study that designed and evaluated a behavior-centered approach to improving breastfeeding-friendly practices of primary health care providers in Kosovo. Only the scoring guide related to the questions within the scope of the validation study is presented below.

1. Clinical Skills: All Mothers

Question	Description
1. Discuss breastfeeding or infant feeding.	Score YES if staff or mother talk at all about infant feeding or how baby is being fed.<br><br>Score NO if staff or mother did not raise this topic.
2. Explain the benefits of breastfeeding	Score YES if staff explain along the following lines:<br>• Breastmilk is the ideal food for baby and provides 100% of its nutrition for the first six months. ("…and it continues to provide up to half or more of a child's nutritional needs during the second half of the first year, and up to one-third during the second year of life."[41])<br>• Supplementing with other liquids or foods will: 1) lessen mother's milk supply; 2) baby will not get the same level of protective benefits; 3) reduce baby's nutrient intake and for solids there is increased risk of choking, obesity and upset stomach.<br>• Talk about complementary feeding as follows:<br>• Timely (start from 6 months age), adequate (amount, frequency and variety based on nutritional needs at each age), safe (preparation to avoid contaminants) and appropriate (correct consistency for age and given according to 'responsive feeding' principals).<br>• Continued breastfeeding is important as "…it continues to provide up to half or more of a child's nutritional needs during the second half of the first year, and up to one-third during the second year of life."[41] Continued breastfeeding also provides a range of benefits beyond its nutritional value for babies (better protection from disease, SIDS, allergies, diabetes and obesity), for mother (better protection against breast and ovarian cancers and osteoporosis, helps with weight loss, lowers risk of PPD, saves time and money) and for both (increased bonding share, providing opportunities to share love, comfort and reassurance).<br><br>Score NO if staff did not raise this topic or if staff inadequately explain importance or advised complementary feeding before six months.
3. Explain a woman's physiological ability to breastfeed	Score YES if staff:<br><br>• Describe that nearly all women can breastfeed and that there are very few medical reasons *not* to breastfeed. Staff might also discuss some reasons why women might think they can't breastfeed (mother's illness/medications, smoking/alcohol, pregnancy, inverted nipples) and give her facts. Staff might ask if mother has heard any other reasons and dispel any myths.<br><br>Score NO if staff did not discuss a woman's physiological ability to breastfeed or if staff provided incorrect information
4. Ask mother if she had any breastfeeding questions or concerns	Score YES if staff asked mother if she had any questions or concerns about breastfeeding.<br><br>Score NO if staff or patient do not raise this topic.

5. Explain follow up visits required	Score YES if staff gave mother information if/when a follow up visit was required.<br><br>Score NO if staff or mother did not raise this topic.
6. Inquire about mothers' support structure	Score YES if staff asks directly or has any conversation about how the mother's family or friends feels about breastfeeding and if she thinks they will be supportive.<br><br>Score NO if staff did not raise this topic.
7. Give take-home material about breastfeeding	Score YES if staff gives mother take home information about breastfeeding e.g. brochure.<br><br>Score NO if no take-home information is given.
8. Explain breastfeeding support resources available	Score YES if staff gave mother any information about where to get more advice or support for breastfeeding, either proactively or in response to a mother's question.<br><br>Score NO if staff or patient did not raise this topic.
9. Promote breastmilk substitutes (formula)	Score YES if staff promoted or provided any breastmilk substitutes (formula). This does not include responding to a mother's question about formula: staff should respond to any questions with factual information, however if when responding they appear to promote formula the score should be YES.<br><br>Score NO if staff did not raise this topic or responds to a mother's question with only factual information and reaffirms that breastmilk is the ideal food for baby.
10. Observe mother breastfeeding	Score YES if staff observed or asked to observe mother breastfeeding.<br><br>Score N/A if baby is not present with the mother during the visit.<br><br>Score NO if staff did not observe or ask to observe mother breastfeeding when there was a clear need to i.e. if mother was having problems breastfeeding.

2. Interpersonal Skills

The rating scale for each of the following skill components is as follows:

*<u>1 = not at all.</u> The staff do **none** of the behaviors in the "Description" column and this component needs a great deal of improvement.*

*<u>2 = a little</u>. The staff do **a small number** of the behaviors in the "Description" column and this component needs a lot of improvement.*

*<u>3 = a moderate amount</u>. The staff do **some** of the behaviors in the "Description" column and this component needs a moderate amount of improvement.*

*<u>4 = a lot</u>. The staff do **the majority** of the behaviors in the "Description" column and this component needs a little improvement.*

*<u>5 = a great deal</u>. The staff do **all** of the behaviors in the "Description" column and this component needs no improvement.*

**Question**	**Description**
11. Really listens to woman and understands concerns	• Asks open questions: these usually start with "How? What? When? Where? Why?" e.g. "How is breastfeeding going for you?".<br>• Shows interest e.g. uses gestures such as nodding and smiling, and simple responses e.g. "Mmm", or "Aha".<br>• Reflects back what the mother says i.e. repeating back what a mother has said to show she has been heard, and to encourage her to say more e.g. if a mother says: "My

	baby was crying too much last night." The staff could say: "Your baby kept you awake crying all night?"
12. Makes woman feel comfortable to express opinions/feelings/concerns	• Accepts what a mother thinks and feels.<br>• Staff responds to mother's opinions, feelings and/or concerns in a neutral way, and does not agree or disagree.<br>• Staff use skills such as reflecting back, showing interest and empathizing.
13. Explains things well and gives practical help	• Gives practical help e.g. tells mother things that she can do today, not in a few weeks. This might not be directly breastfeeding related but will support it e.g. when a mother feels tired give her ideas for getting more rest.<br>• Gives a little relevant information e.g. staff gives only one or two pieces of information at a time.<br>• Uses simple familiar terms to explain things to mothers and does not use technical medical terms.<br>• Gives information in a positive way, so that it does not sound critical.

General Observations

In the General Observations field:

- Note if the mother (or anyone accompanying her) was the first one to raise the issue of infant feeding.
- Describe any visual aids that were used in answering questions, describing aspects of breastfeeding or overcoming problems.
- Briefly note the power relationships and dynamic between staff and mother (and anyone with patient e.g. husband, grandmother). Did the staff play out their role as expected i.e. caring medical provider, authority position? Did the staff express an obvious attitude (positive or negative) towards the mother in general or the topic of breastfeeding, and if so describe?
- Note if COVID-19 precautions (masks and physical distancing) appeared to impact the interaction between staff and patient. Are staff adapting to compensate e.g. are they doing anything different to pre-COVID times? Should staff have observed breastfeeding but did not? Do mothers appear less receptive to messages or assistance?)
