# Supplementary material for: Assessing the validity of maternal report on breastfeeding counselling in Kosovo’s primary health facilities
Source: BMC Pregnancy Childbirth. 2024 Aug 27;24:558. doi: 10.1186/s12884-024-06766-8 (PMC11348650; doi:10.1186/s12884-024-06766-8)
Supplement: Supplementary file 7 — Supplementary Material 7 [file 12884_2024_6766_MOESM7_ESM.pdf]

Additional File 7 - Comparison of validation results for the two sample periods

	Consultation Observations (Ref Standard)		Exit Interviews with Mothers		Matched Pairs											
	Prevalence (95% CI)		Prevalence (95% CI)		Matched Pairs (N)		Agreement (%)		5 Counts Per Cell?		Sensitivity (95% CI)		Specificity (95% CI)		AUC (95% CI)	
**INDICATOR**	2019	2021	2019	2021	2019	2021	2019	2021	2019	2021	2019	2021	2019	2021	2019	2021
**1. CLINICAL BEHAVIORS**																
Provider discussed breastfeeding or infant feeding.	87.4 (83.4-91.3)	92.8 (90.0-95.6)	86.3 (82.2-90.4)	86.7 (83.1-90.4)	277	332	82%	91%	Y	Y	89.3 (84.7-92.9)	91.9 (88.3-94.7)	34.2 (19.1-52.2)	79.2 (57.8-92.9)	0.62 (0.54-0.70)	0.86 (0.77-0.94)
Provider explained the benefits of breastfeeding.	60.7 (54.7-66.7)	58.1 (52.7-63.5)	55.0 (49.0-61.0)	60.8 (55.5-66.1)	245	324	69%	75%	Y	Y	70.4 (62.5-77.5)	80.5 (74.2-85.9)	67.7 (57.3-77.1)	66.4 (57.8-74.3)	0.69 (0.63-0.75)	0.73 (0.69-0.78)
Provider explained a woman's physiological ability to breastfeed.	36.5 (30.5-42.5)	40.8 (35.5-46.1)	9.2 (5.6-12.8)	51.5 (46.1-57.0)	229	323	62%	71%	Y	Y	13.5 (7.2-22.4)	76.9 (68.8-83.7)	92.9 (87.3-96.5)	66.1 (58.9-72.8)	0.53 (0.49-0.57)	0.72 (0.67-0.76)
Provider asked mother if she had any breastfeeding questions or concerns.	36.2 (30.1-42.2)	66.8 (61.2-72.4)	21.8 (16.7-27.0)	46.0 (40.5-51.4)	225	270	68%	73%	Y	Y	38.4 (28.1-49.5)	69.4 (62.2-76.1)	87.1 (80.3-92.1)	81.1 (71.5-88.6)	0.63 (0.57-0.69)	0.75 (0.70-0.81)
Provider explained follow-up visits required.	93.8 (91.0-96.7)	95.7 (93.5-97.9)	58.0 (52.1-63.9)	78.6 (74.1-83.1)	267	319	59%	83%	Y	N	59.0 (52.6-65.1)	83.3 (78.7-87.3)	56.3 (29.9-80.2)	84.6 (54.6-98.1)	0.58 (0.45-0.71)	0.84 (0.74-0.94)
Provider inquired about mothers' support structure.	13.1 (8.9-17.4)	16.6 (12.6-20.6)	5.3 (2.5-9-8.1)	6.0 (3.3-8.6)	224	318	83%	82%	N	Y	9.4 (2.0-25.0)	14.8 (6.6-27.1)	95.8 (92.0-98.2)	95.8 (92.7-97.9)	0.53 (0.47-0.58)	0.55 (0.50-0.60)
Provider gave take-home material about breastfeeding.	5.1 (2.4-7.8)	16.4 (12.4-20.4)	1.6 (0.0-3.1)	15.3 (11.3-19.3)	232	318	93%	89%	N	Y	0.0 (0.0-24.7)	64.2 (49.8-76.9)	98.2 (95.4-99.5)	94.3 (90.8-96.8)	0.49 (0.48-0.50)	0.79 (0.73-0.86)
Provider explained breastfeeding support resources available.	5.9 (3.0-8.8)	26.2 (21.4-31.0)	6.0 (3.0-8.9)	22.4 (17.8-27.0)	230	313	88%	80%	N	Y	0.0 (0.0-23.2)	55.4 (44.1-66.3)	93.5 (89.4-96.4)	89.1 (84.4-92.8)	0.47 (0.45-0.48)	0.72 (0.67-0.78)
Provider did not promote breastmilk substitutes.	98.8 (97.4-100.2)	93.1 (90.3-95.8)	98.0 (96.2-99.7)	98.1 (96.6-99.6)	221	320	96%	92%	N	N	97.7 (94.7-99.3)	98.3 (96.1-99.5)	0.0 (0.0-70.8)	4.3 (0.1-21.9)	0.49 (0.48-0.50)	0.51 (0.47-0.56)
Provider observed mother breastfeeding.	11.4 (7.5-15.3)	16.9 (12.8-21.0)	5.7 (2.8-86.1)	20.7 (16.2-25.1)	235	318	86%	92%	Y	Y	17.2 (5.8-35.8)	87.3 (75.5-94.7)	96.1 (92.5-98.3)	93.2 (89.4-95.9)	0.57 (0.50-0.64)	0.90 (0.86-0.95)

	Consultation Observations (Ref Standard)		Exit Interviews with Mothers		Matched Pairs											
	**Prevalence (95% CI)**		**Prevalence (95% CI)**		**Matched Pairs (N)**		**Agreement (%)**		**5 Counts Per Cell?**		**Sensitivity (95% CI)**		**Specificity (95% CI)**		**AUC (95% CI)**	
**INDICATOR**	**2019**	**2021**	**2019**	**2021**	**2019**	**2021**	**2019**	**2021**	**2019**	**2021**	**2019**	**2021**	**2019**	**2021**	**2019**	**2021**
**2. INTERPERSONAL BEHAVIORS***																
Provider really listened to, and understood the concerns of, mother.	14.3 (10.1-18.5)	57.8 (52.5-63.2)	66.5 (60.8-72.2)	80.7 (76.4-84.9)	255	331	38%	65%	Y	Y	68.6 (50.7-83.1)	89.5 (84.3-93.5)	33.2 (27.0-39.8)	31.4 (23.9-39.8)	0.51 (0.42-0.59)	0.60 (0.56-0.65)
Provider made mother feel comfortable to express her opinions, feelings and concerns.	14.7 (10.4-18.9)	58.1 (52.8-63.5)	66.5 (60.8-72.2)	79.0 (74.6-83.4)	255	329	38%	66%	Y	Y	67.6 (50.2-82.0)	89.0 (83.7-93.1)	32.6 (26.4-39.2)	34.8 (26.9-43.4)	0.50 (0.42-0.58)	0.62 (0.57-0.66)
Provider explained things well and gave practical help in a way mother could understand.	11.9 (8.0-15.8)	44.9 (39.5-50.3)	34.2 (28.4-40.0)	47.7 (42.3-53.1)	254	331	68%	75%	Y	Y	58.6 (38.9-76.5)	75.0 (67.2-81.7)	68.9 (62.4-74.9)	74.3 (67.4-80.5)	0.64 (0.54-0.73)	0.75 (0.70-0.79)

**Likert scale converted to binary result by collating "A great deal" and "A lot" into "Yes" and "Not at all", "A little" and "A moderate amount" into "No".*
